# Supplementary material for: Heritability of Protein and Metabolite Biomarkers Associated with COVID-19 Severity: A Metabolomics and Proteomics Analysis
Source: Biomolecules. 2022 Dec 27;13(1):46. doi: 10.3390/biom13010046 (PMC9855380; doi:10.3390/biom13010046)
Supplement: Supplementary file 1 [file biomolecules-13-00046-s001.zip › biomolecules-2073182-supplementary.pdf]

	inosine	N-palmitoyl-D-erythro-sphingosine	maltose	2-hydroxybutyrate (AHB)	myo-inositol
inosine	1.00				
N-palmitoyl-D-erythro-sphingosine	0.27	1.00			
maltose	0.33	-0.09	1.00		
2-hydroxybutyrate (AHB)	0.12	0.14	0.10	1.00	
myo-inositol	0.28	-0.06	0.24	0.10	1.00

Supplemental Figure S1: Correlation matrix of metabolites upregulated in COVID-19 disease states.

Metabolites that were upregulated in non-severe and severe COVID-19 in the [1] dataset were identified in our erythrocyte dataset and Pearson correlations were calculated among all metabolites.

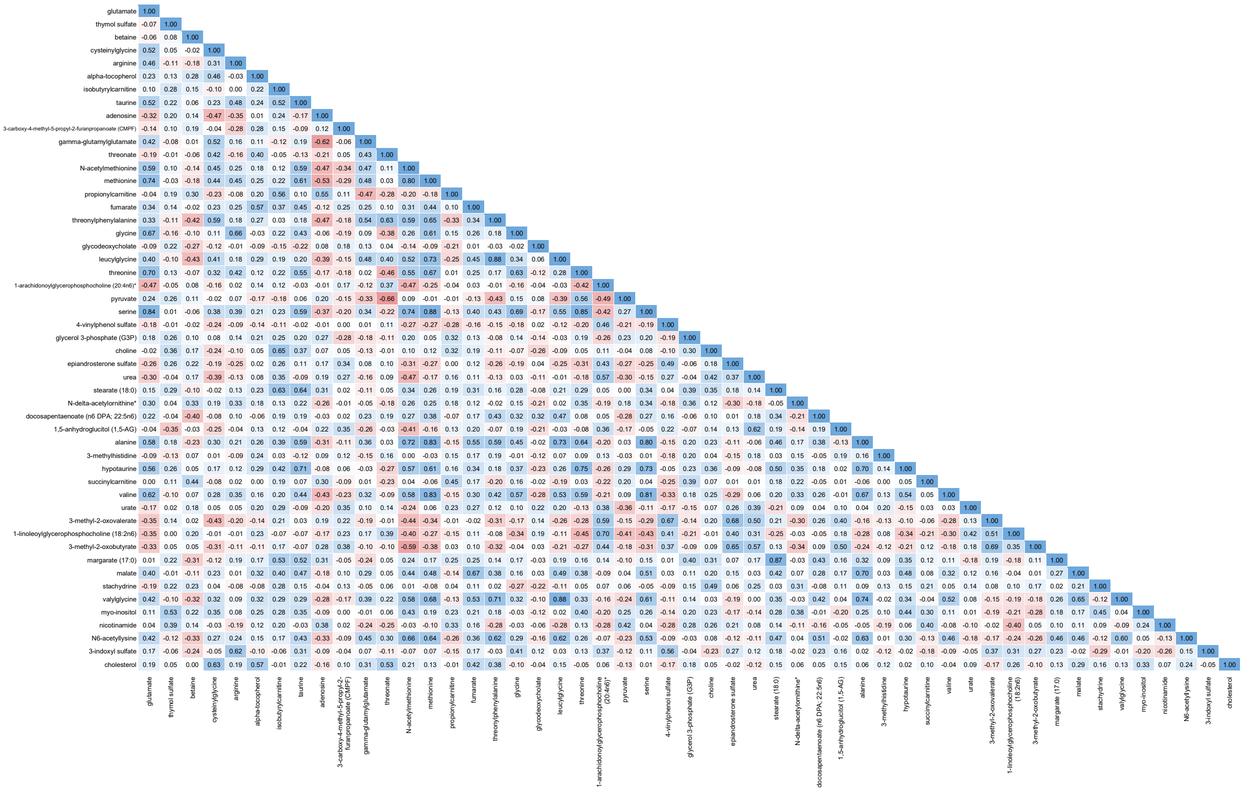

Supplemental Figure S2: Correlation matrix of metabolites downregulated in COVID-19 disease states.
Metabolites that were upregulated in non-severe and severe COVID-19 in the [1] dataset were identified in our erythrocyte dataset and Pearson correlations were calculated among all metabolites.

Reference

1. Shen, B.; Yi, X.; Sun, Y.; Bi, X.; Du, J.; Zhang, C.; Quan, S.; Zhang, F.; Sun, R.; Qian, L.; et al. Proteomic and Metabolomic Characterization of COVID-19 Patient Sera. *Cell* **2020**, *182*, 59–72.e15.
